# Updates on Treatment of Attention-Deficit/Hyperactivity Disorder: Facts, Comments, and Ethical Considerations

**DOI:** 10.1007/s11940-012-0197-2

**Published:** 2012-09-12

**Authors:** Aribert Rothenberger, Lillian Geza Rothenberger

**Affiliations:** 1 Child and Adolescent Psychiatry, University Medicine Göttingen, von-Siebold-Str. 5, 37075 Göttingen, Germany; 2 Institute of Ethics and History in Medicine, Center for Medicine, Society and Prevention, University of Tuebingen, Tuebingen, Germany

**Keywords:** Attention-deficit/hyperactivity disorder, ADHD, Children, Adolescents, Treatment, Drugs, Behaviour therapy, Ethics, Ethical considerations

## Abstract

Within the last 2 years the bulk of information on evidence based treatments in ADHD was reviewed quite intensively and new empirical studies could be added. This update reports comprehensively about actual and essential facts in the field related to brain development and sensitive periods, predictors of treatment, safety of medication, value of naturalistic studies, new drugs and complementary medicine, behavioral interventions including neurofeedback and psychosocial treatment, treatment of comorbidity, and ethical considerations including preventive aspects. The updated combination of well selected evidence based treatments (ie, pharma plus non-pharma) seems to be clinically and ethically recommended as also suggested by the European and American guidelines on ADHD.

## Introduction

### A bulk of empirical information

Attention deficit hyperactivity disorder (ADHD) is one of the most common (worldwide prevalence of about 5 %) [1]; and debilitating child psychiatric disorders with the core symptoms of inattention, hyperactivity, and impulsivity. Also a high frequency of co-existing developmental psychopathological conditions (eg, anxiety, depression, tics, autism, and oppositional behavior) may lead to psychosocial impairment and often need to be treated. Treatment programs usually include medication and psychosocial interventions [2].

From the year 2011 to 2012, several reviews and meta-analyses covered the field of treatment options in ADHD and their degree of empirical evidence (eg, [3•, 4••, 5, 6••, 7•, 8•, 9] European ADHD Guidelines Group [EAGG] submitted). Also, the actual clinical practice guidelines for ADHD of the American Academy of Pediatrics appeared in 2011 [10••].

Hence, this review intends to comment on the conclusions of the reviews and give an update on some neglected but important issues of pharmacological and non-pharmacological treatment options of ADHD like prevention and ethics.

## Treatment

### Neurodevelopmental aspects of interventions

Whatever the treatment for ADHD is, to be successful it must change the function (and probably also the structure) of the child’s brain. Within the last few years we learned from experiments of ADHD on animal models that stimulants may bring the increased density of dopamine transporters back to a normal range [11]. Moreover, in an Annual Research Review on developmental neuropharmacology, Andersen and Navalta [12•] reported systemically how currently used psychotropic agents in ADHD (like stimulants and atomoxetine) might influence long-term brain maturation; especially, when given during sensitive periods like pre-puberty. This is supported by Roessner et al. [11] for methylphenidate and an animal study on reboxetine by Bock et al. [13]. Further investigations are necessary before one might think of early drug intervention in ADHD in order to stop its progression and prevent symptoms from manifesting in the long-run.

However, some recent clinical investigations give hope. For example, Hoekzema et al. [14] assessed “whether a cognitive training program applied to ADHD patients can contravene some of the associated neuroanatomical alterations”. Indeed, they observed MRI volumetric increase in frontal cortex and cerebellum concluding that cognitive training induced neuroanatomical plasticity. Further, Nakao et al. [15] conducted a voxel-based meta-analysis comprising 278 patients with ADHD and 344 healthy subjects (children and adults) exploring the association of stimulant medication and gray matter volume. Their positive finding in the basal ganglia suggests that ADHD patients may progressively catch up with their developmental delay, especially when they are treated with stimulant medication.

In summary, pharmacological as well as non-pharmacological interventions in ADHD seem to support developmental normalization and/or compensation of brain alterations in these patients. The chance may be highest during sensitive phases of neuronal plasticity. Thus there might be a potential for secondary prevention in case of early detection of ADHD.

### Predictors of treatment

A very early start of treatment should qualify for a safe use. Unfortunately, clear predictors for this and successful symptom reduction are lacking. At the moment pharmacogenetic approaches are en vogue, but before the field can offer data to be used for personalized ADHD treatment, pharmacogenetic studies with larger samples and range of outcomes related to efficacy, effectiveness, and safety are needed to determine the utility of genomic information [16]). Their data and methodological limitations allowed only a rough conclusion, namely “that DAT and DRD4 polymorphisms may be associated with individual variability in MPH dose-response”. Markers of the dopaminergic system may be the most promising candidates for a personalized pharmacogenetic approach to date, but such a pharmacologic management of ADHD is not near.

Concerning non-genetic predictors, probably the prepotent response inhibition reaction time in a neuropsychological Stop-Signal-Task seems a good candidate. Children with lower levels of inhibition showed worse outcome after 10 weeks of MPH treatment, independent of dose [17].

Finally, success of neurofeedback training (NF) is the better the higher the pre-NF-training amplitude of the contingent negative variation (CNV); the latter is an event-related electrophysiological brain potential generated mainly by anterior cingulate cortex [ACC]) [18].

Hence, preparation for and inhibition of a goal directed behavior is related to the dopaminergic genes [19] and thus indicators of the latter should be evaluated further as predictors for treatment in ADHD.

### Medication and safety

Treatment options for ADHD are always lively debated in light of probable side effects, especially if medication with stimulants is considered. Fortunately, guidelines help clinicians to provide high-quality care to their patients by delivering evidence-based treatment programs.

The European ADHD Guidelines Group (EAGG) reviewed the published literature on adverse effects of the drugs that are licensed in Europe in order to treat ADHD [20••] (an updated and very practical version of this topic will appear soon and comes to a similar conclusion). They reported on cardiac adverse events, suicide-related events, growth in childhood, sleep disturbances, tics and Tourette’s syndrome, substance abuse and misuse and diversion, epilepsy and seizures, psychotic symptoms, drug holidays, sudden death, liver-failure, and long-term changes in the brain. They conclude, that “for many clinicians, the balance of risks against possible benefits of treatment will be seen as favorable in most cases”.

Especially, because of critical reports, cardiovascular risks were investigated and reviewed very thoroughly [21–25]. Stimulants are increasing noradrenergic and dopaminergic transmission and thus via sympathomimetic effects increase blood pressure of about 5 mm Hg and heart rate of about 10 beats/minute. Usually, this does not change electrocardiographic parameters and risk for cardiovascular events. However, about 5 %–15 % of children may show greater increases and/or report cardiovascular complaints [23]. Therefore, to prevent adverse events, the physician should follow the clinical recommendations of societies and guideline groups for screening and monitoring of cardiovascular disease/complaints in children and adolescents treated with stimulants and noradrenergic drugs like atomoxetine, clonidine, and guanfacine. In cases of uncertainties an assessment by and shared-care with a pediatric cardiologist is recommended [24]. This is in line with a recent public health study on private insurance data of 171,126 6–21 year old subjects without known cardiovascular risk factors. Clinical diagnosis of cardiovascular events and symptoms were rare and not associated with stimulant use for an average follow-up of 1 year 9 months [22]. Also, Vitiello et al. [25] conducted a secondary analysis of the MTA-study on blood pressure and heart rate over 10 years. No symptomatic cardiovascular event leading to medical attention was reported. They conclude that stimulant treatment did not increase the risk for prehypertension or hypertension, not even in a subset of the patients. The effect on heart rate was driven by current use of stimulants reflecting chances for reversibility to normal. All in all, reviews and new data give evidence for a detectable adrenergic cardiovascular effect even after years of treatment with stimulants, but with careful monitoring this should not lead to clinical problems. However, in order to further elucidate the long-term cardiovascular and other effects a publicly funded ADHD Drug Use Chronic Effects (ADDUCE) study was recently launched in Europe.

A second important and still debated point of safety is children’s growth. Dura-Trave et al. [26] measured weight and height in 187 ADHD children treated with OROS-MPH (about 8 years of age at the beginning) during 4 years of follow-up. Mean weight and height values (in absolute terms) increased slightly over time (14.9 kg and 19.8 cm) reaching age appropriate group values for both weight and height after 48 months. However, it remains to be discussed if an observed transitory growth deficit might be best explained by transitional nutritional aspects, which may disappear under the long-term treatment with stimulants and thus the final stature measures may be reached. Since these are group effects, some children may not show such a spontaneous recovery. Hence, careful monitoring, nutritional optimization, and drug holidays should be considered within an individual/personalized drug treatment plan for prevention of final weight and height deficits.

### Value of naturalistic studies

Randomized controlled trials (RCTs) are the gold standard in order to clarify the efficacy of an intervention. Post-RCT observational studies are required to ensure well-monitored, up-to-date treatment management regarding effectiveness (“is it of use?”), and efficiency (“how much benefit at what cost?”), especially for a long-term perspective [27]. Today, collecting, reporting and interpreting data from naturalistic studies is still heterogeneous. It follows, that the value of such urgently needed studies is mixed.

Concerning pharmacological treatment of ADHD observational studies contributed a great deal to the improvement of daily clinical practice. Although it is not yet clear from the naturalistic part of the MTA-study, what treatment contributed what in the long run [17, 28] some more recent observational trials could indicate that switching from one MPH preparation to another, either from MPH-IR to MPH-MR or even from one MPH-MR formulation to another, appears to be a valid clinical approach. The improvement might be best explained by (1) increased/optimized dose of MPH, (2) shorter intervals between visits, (3) positive expectation of improvement, and (4) improvement of adherence in the long-term [27].

Finally, van der Oord et al. [17] pointed out that in a naturalistic follow-up of 24 ADHD children over 4.5 to 7.5 years treatment with behavior therapy next to MPH may not have additive effects on short-term outcome, but may cause long-term effects, ie, less methylphenidate use in adolescence. If this is proven to be true by future evidence, such a combined treatment might reflect, economically and ethically, an improvement of ADHD treatment, including the prevention of side effects by medication.

### Drugs and complementary medicine

There is no break-through for treatment of ADHD expected within the short-term, but the new recommendations of the American Academy of Pediatrics [10••] are helpful and quite close to the European Guidelines [2, 28], ie, for preschool children (4–5 years of age) behavior therapy is first-line, and MPH second-line; for elementary school-aged children (6–11 years of age) both, behavior therapy and/or medication should be prescribed—preferably both. Drugs recommended are stimulants, atomoxetine, extended-release guanfacine, and extended-release clonidine (in that order). For adolescents (12–18 years of age) recommendations are similar and assent of the youngster is underlined.

In clinical practice, the different drugs in use are sometimes administered adjunctively, without having a controlled evidence base for this. For the combined application of guanfacine XR plus stimulants, Wilens et al. [29] showed a significantly greater improvement over placebo plus stimulant in ADHD symptoms and generated no new safety signals. A drug not yet mentioned in the guidelines but already in clinical use and promising is lis-dexamfetamine (LDX). LDX is a pro-drug, which means that lysin is pharmacologically coupled with dexamfetamine and absorbed as the combination. In the blood both parts are metabolically separated by enzymatic hydrolysis and only now dexamfetamine can act at the neuronal cells as a stimulant. Due to the rate-limiting nature of hydrolysis, the toxicity potential, and risks of overdose are reduced. This special situation decreases the risks for misuse and diversion of this pro-drug [30]. Two recent studies [31, 32] inform us that LDX seems to be a safe and efficacious treatment for symptom relief in ADHD. All doses (30, 50, 70 mg) in the short-term (4–5 weeks) were efficacious vs placebo and demonstrated an acceptable safety profile as already known from other LDX-studies.

For the practitioner it would be important to know from the beginning, what stimulant or noradrenergic drug may be the best one to support the patient. This predictive approach is limited since, so far, individual predictors (like CNV and certain gene-profiles) only predict treatment outcome in general, but not for a specific drug or intervention. Also, recent comparisons of Medikinet-R vs Concerta [33] and between extended-release dexmethylphenidate vs extended-release mixed amphetamine salts [34], as well as a meta-analysis of MPH vs atomoxetine [5] came to similar/non-inferior efficacy, and acceptability when similar doses were given. This weakens the individualized predictive approach and supports the recommendations of the existing clinical guidelines.

Finally, co-existing sleep problems in ADHD may complicate family interaction at home and increase sleepiness during the day. So far, melatonin is often used off-label in order to reorganize the disturbed circadian rhythm. A first RCT on the effect of l-theanine on objective sleep quality (using actigraphy) in 98 ADHD boys aged 8–12 years demonstrated that 400 mg daily of l-theanine might improve some aspects of sleep quality but more rigorous studies are needed.

Complementary medicine in ADHD (electrolytes, restriction diet, food color additives, and omega-3 fatty acid) is again in the focus of ADHD treatment options.

While some news about zinc and iron [35, 36•] needs to be better substantiated, a meta-analysis on restriction diet and synthetic food color additives offers some helpful and clarifying evidence [6••]. Within the last years, subsequent studies appeared leading to divergent conclusions on the risks of food colors and the practical value of restriction diets. Overall, Nigg et al. [6••] drew a mixed conclusion: “Although the evidence is too weak to justify action recommendations, absent a strong precautionary stance, it is too substantial to dismiss” (p. 96). An estimated 8 % of children with ADHD may have symptoms related to synthetic food colors, some of which being responsive to restriction diet. However, effect sizes can be found only between 0.12 and 0.27 (depending on the source of information like parents, teacher, physician, attention-test) and are far behind that what is known from medication (about 0.7–0.9) and behavior therapy (about 0.5). One should remain critical against restriction diet, although a strictly supervised restriction elimination diet may be a valuable instrument to assess whether ADHD is induced by food [37•]; (remark: this study was removed as outlier by Nigg et al. [6••] because of weak blinding and unexpected high effect sizes). A recent review on the role of dietary methods for ADHD focuses on efficacy and practice while concluding that additive-free and oligoantigenic/elimination diets are time-consuming and disruptive to the household [38].

Hence, adherence problems are foreseeable even if indication is given only in selected patients, contradicting Pelsser et al. [37•] who “think that dietary intervention should be considered in all children with ADHD” (p. 502).

When talking about complementary medicine in ADHD, omega-3 fatty acid supplementation is always a favorite [7•, 36•]. The latter authors focused on omega-3 with a systematic review and meta-analysis. Overall, the 10 trials included (*n* = 699 patients) showed a small but significant constant effect size (SMD = 0.31). However with the better investigated, more effective, and approved psychopharmacological agents like stimulants and atomoxetine at hand, only an augmentation seems reasonable. Because of poor quality and weak blinding in earlier clinical studies, further trials each involving at least 330 children with ADHD are needed to demonstrate efficacy with an effect size of about 0.3.

### Behavioral interventions

In their meta-analytic review on non-pharmacological treatments for ADHD Hodgson et al. [8•] reported a total of 15 controlled treatment studies conducted between 1994 and 2009. Their latest references stem from 2008 and it remains unclear, why their publication dated of 2012 neglects the last 3.5 years of research in the field. The review is further limited by the small number of studies for each kind of treatment (between 1 and 4) and not excluding other studies than RCTs. Nevertheless, the evaluation might give a first critical impression of the basic value of non-pharmacological treatments for ADHD. To update this impression, the more timely and already submitted meta-analysis of the Eunethydis ADHD Guidelines Group is urgently warranted (EAGG submitted).

Considering the overall effect on the range of the reported 20 outcome measures examined, (eg, ADHD symptoms, other behavior, neuropsychological test performance, IQ, academic ability). Hodgson et al. [8•] favored neurofeedback as the most efficacious (average weighted effect size of 0.09), while behavioral modification, working memory training, school-based treatment, parent training, and self-monitoring did not show positive average weighted effect sizes across the outcome measures, reflecting no generalized greater improvement in the treatment group vs the control group, and thus might not be deemed efficacious for all parameters investigated.

However, the main purpose of behavioral interventions is to modify behavior and self-regulation. From this point of view Hodgson et al. [8•] assume, that mainly behavior therapy and, as a special technique within this area, neurofeedback may be recommended to treat ADHD.

Two recent narrative reviews confirm the recommendation of neurofeedback for ADHD on the basis of the increasing evidence for its short-term clinical efficacy and stability of treatment effects on ADHD symptoms for at least 6 months [39••, 40].

Working memory training is another neurotraining but without controlling for brain activity. A meta-analytic review on 23 studies concluded that these programs produce short-term improvement in working memory skills, which do not generalize to other neuropsychological functions or clearly to ADHD symptom-reduction. Therefore, this review cast doubt on its clinical relevance in ADHD [3•].

Since there exist already a multitude of reviews and meta-analyses on psychosocial treatments for ADHD, the purpose of the systematic appraisal of the evidence by Watson et al. [4••] was to evaluate their quality in order to refine existing recommendations. Including 13 systematic reviews and 8 meta-analyses they used the general term of “psychosocial treatments” to subsume, eg, cognitive-behavioral interventions directly with the child, parent training, behavior modification by teacher, family counselling, self-regulation methods, EEG-Biofeedback, and a combination of psychosocial treatments. The highest rated quality articles noted that pharmacologic plus cognitive-behavioral intervention (ie, multimodal treatment) had the best benefit for ADHD behaviors and academic and social outcomes.

Two Cochrane reviews, one on parent training [41•] and another one on social skills training [42] added to this conclusion, that parent training might have a positive effect on the behavior of children with ADHD (especially preschool children), but the evidence from this review is not strong enough to form a basis for clinical practice guidelines. For social skills training there is little evidence (ie, no statistically significant treatment effects on social skills competence, general behavior or ADHD symptoms) and lacking of non-biased data. This leaves it open what to recommend.

Further studies which try to test combined parent and teacher/child psychosocial treatment programs are ongoing with some preliminary success mainly on children’s externalizing behavior [43, 44]). With all these psychosocial treatments in mind it seems highly important to consider the influence of parental ADHD [45] and, related to this, also the genetic background of the child and the parents [46].

### Treating comorbidity

In ADHD, comorbidity is the rule (60 %–80 %) and, psychosocially, the most impairing disorder needs to be treated first, but sometimes the co-occurring problems need to be treated in parallel [47]. The guidelines of the American Academy of Pediatrics ([10••], (p. 1019) state: “The effect of co-existing conditions on ADHD treatment is variable. In some cases treatment of the ADHD resolves the co-existing condition. For example, treatment of ADHD might resolve oppositional defiant disorder or anxiety. However, sometimes the co-occurring condition might require treatment that is in addition to the treatment of ADHD” (eg, neuroleptics for tic disorders; SSRI for depression; behavior therapy for obsessive-compulsive disorder, or substance use disorder). There is no question that in the case of comorbidity ADHD symptoms may be successfully treated with stimulants or atomoxetine or clonidine [48, 49]. Clinical practice reflects that use of psychotropic medication for ADHD with co-existing disorders is wide spread. Frazier et al. [50] reported of the first wave of a USA-wide representative study of adolescents aged 13–17 in special education. Youths with autism spectrum disorder (ASD) plus ADHD had a rate of 58 %, those with ADHD-only 49 % and youths with ASD-only 34 %. As expected, in patients with ADHD-only stimulants were dominant, while the 2 ASD-groups used medication across a variety of medication classes depending on associated symptoms. In about 30 % of comorbid cases more than 2 medications were taken. This raises 2 unresolved questions. First, the evidence is low for studies with combined medication. For example, in a secondary analysis of a study with ADHD in adults, Biederman et al. [51] concluded that they found no moderating effects of concomitant use of antidepressants on the ADHD treatment effect with OROS-MPH. Second, a paucity of data exists also for the evidence related to the combined use of medication and psychotherapy (eg, [52]).

In summary, studies on efficacy, effectiveness, and safety of poly-pharmacy in ADHD comorbidity are needed to provide a better empirical guideline for the widespread use of multiple medications, often in combination with psychotherapy.

### Ethical considerations

There exists an ongoing ethical discussion about how the physician should handle treatment when sharing and selling of prescribed stimulants may play a role. Especially, when these drugs are used to treat patients with ADHD plus a substance use disorder (SUD). Diversion of stimulants varies across adolescent populations and shows the highest prevalence among ADHD patients using other illicit drugs [53]. Fortunately, “concerns that these practices have become more prevalent as a result of increased prescribing are not supported by large-scale population surveys” [53]. Further, with close monitoring stimulants can be safely used to treat ADHD and reduce co-existing substance abuse. However, since prescription stimulants diverters have more childhood conduct problems than non-diverters, this area of developmental psychopathology needs special attention within a treatment program for ADHD plus SUD including stimulant prescription [54, 55].

Another point of on-going ethical controversy is a probable pharmacological neuroenhancement (doping for performance) in ADHD. Neuroenhancement (and probably related to this faking ADHD) is merely a problem of adults [56–58], especially in cases where the diagnostic criteria according to DSM-IV and ICD-10 are not or not completely fulfilled but symptom-related psychosocial impairment hinders the child’s development.

This discussion includes different views on ADHD concerning, eg, inclusion/exclusion criteria, the question of dimensionality vs categorization, validity, and reliability of diagnostics in clinical practice and, thus, the sound empirical and practical basis for clear treatment decisions in contrast to hidden neuroenhancement within a grey zone of diagnostics [59].

Ethical problems and controversies could be minimized if a normalization of Quality of Life in patients with ADHD and their families [60] would be accepted as the goal for treatment decisions. The latter should be made along the ADHD guidelines available [2, 10••] and should include a critical evaluation and monitoring of the psychosocial context in order to avoid medication of problems, which may be solved otherwise.

ADHD can be a lifelong threat starting early in life with long-term treatments of different kind. Unfortunately, medication nonadherence seems to be common in childhood/adolescent ADHD [61]; thus, it is an ethical challenge for the physician to improve compliance within the process of diagnosis and treatment. Therefore, it is important that the child/adolescent is always involved in the assent/informed consent process as fully as possible. Such a process, which should be revisited over time, should also have a scientific empirical basis as the treatment itself. Data are scarce and, therefore, the article of Schachter et al. [62] is welcome. They focused on the understanding of information necessary to consent the treatment with stimulant medication. Fifty-eight ADHD and 64 control adolescents (12–16 years of age) and their parents were studied with interviews and questionnaires. Knowledge and, thus, the basic precondition for informed consent were increased after the information session for all subjects. The study has shown that adolescents can gain the capacity to consent after adequate information about benefits and risks of stimulants. Their understanding is similar to that of their parents. Thus, the adolescents` inclusion and reinforcement of their autonomic decision in this consensual process should be regarded.

Close to this process is the ethical impetus to increase shared decision-making in ADHD [63•, 64•]. Shared decision-making (SDM) is ethically recommended in general but it is of particular importance when multiple evidence-based treatments exist and parents, patients, and clinicians value the available options differently as in these situations there is a great potential for conflicts and misunderstanding. Little empirical research is known about the process coming up afterwards and what kind of solution and under which conditions it will be reached. Fiks et al. [65] conducted interviews with 60 parents of children with ADHD (6–12 years of age) and 30 primary care clinicians. Parents and clinicians both viewed SDM favorably, but they understood SDM differently. Pediatric clinicians often tried to persuade families to accept their preferred treatment while parents desired objective and well based information before decision-making, even if they ultimately delegated responsibility for the decision to the clinician [63•, 65]. Such a disparity may influence adherence and revisitation of SDM over time as families acquire real-world experience and new problems have to be discussed. Finally, public health conditions (eg, time and reimbursement available) may modify SDM and its ethical implications. SDM-implications for clinical practice depend highly on such profane issues.

The handling of children’s willingness to use ADHD treatments, medication refusal, and their developmentally adequate self-management of medication are also important issues for ethical considerations, since they are closely related to compliance and adherence, ie, the success of treatment with good psychosocial functioning giving the youngster a better chance for his/her future. This seems to be ethically important with regard to prevention of severe symptoms, adverse events concerning high dosages of medication, and difficulties in social life.

Summarizing the following empirical publications of Bussing et al. [66], Brinkman et al. [67], and Demidovich et al. [68], one can say that the different roles of patients and parents along the lifespan and along the course of ADHD have to be interactively guided by the specialist, (eg,. ensuring a developmentally appropriate transition from family- to self-management of ADHD and its treatment). In particular, adolescents’ perceptions of undesirable effects, like stigma, related to ADHD should be openly discussed [49, 69]. A quality improvement in community-based pediatric ADHD care along the guidelines could further contribute to optimize ADHD interventions [70•].

### Perspectives

Within the last 2 years, the bulk of studies on ADHD treatment could be reviewed to get their essential content and usefulness for further clinical practice including prevention. A broad variety of evidence-based treatments is available like behavior therapy, neurofeedback, family coaching, and psychopharmacology. As the combination of treatments seems to be clinically and ethically recommended, studies of combined treatment are needed to clarify which profile of multimodal treatment fits best the needs of families with an ADHD patient.
